# Vaccine Responses in Early Age

**DOI:** 10.3390/vaccines14070629

**Published:** 2026-07-18

**Authors:** Swetha Parvathaneni, Jiro Sakai, Lunhua Liu, Mustafa Akkoyunlu

**Affiliations:** US FDA/CBER/OVRR/DBPAP, Silver Spring, MD 20993, USA

**Keywords:** neonatal vaccines, T cell-dependent response, T cell-independent response, CD4+ cells, germinal center B cells, T follicular helper cells, adjuvants, innate immune response, cytokines

## Abstract

Neonates and infants exhibit fundamentally distinct immune responses compared to adults, resulting in increased susceptibility to infectious diseases and reduced vaccine efficacy. These age-specific responses are characterized by developmental constraints affecting both adaptive and innate immunity. T cell-independent (TI) responses to polysaccharide vaccines are severely impaired due to reduced transmembrane activator and calcium-modulating cyclophilin ligand interactor (TACI) expression on neonatal B cells, while T cell-dependent (TD) responses are compromised by Th2 bias in the CD4+ T cell compartment, and by restricted T follicular helper (Tfh) cell development. Emerging data suggest that cytokines such as IL-6 have completely opposite effects on Tfh cell development between adults and neonates. Germinal center (GC) B cell responses are further constrained by delayed follicular dendritic cell (FDC) maturation, impaired B cell receptor (BCR) signaling, and elevated frequencies of IL-10-producing regulatory B cells. The overall immunosuppressive phenotype associated with early age extends to the neonatal innate immune system as exhibited by altered dendritic cell (DC) subset distribution, decreased IL-12p70 production, lower expression of MHC class II and co-stimulatory molecules, and increased IL-10 secretion. To overcome these immune constraints, various adjuvants that are shown to enhance immune response to vaccines in adults are considered for early age vaccines. Although some of these adjuvants show promising results in animal experiments, mechanistic studies need to be conducted in detail since adult and neonatal in vivo environments may dictate different outcomes between the two age groups, especially because early-life adjuvant exposure may have long-lasting effects on immune system programming. Elucidation of age-specific immune responses to vaccines and adjuvants will help develop age-tailored strategies to develop safe and effective pediatric vaccines.

## 1. Introduction

The human immune system develops early in fetal life. The emergence of T and B lymphocytes begins in blood and spleen starting from 12 to 14 weeks and 16 to 17 weeks of gestation, respectively [1]. A range of immunosuppressive mechanisms such as regulatory T cells (Treg), hypoxia, and hormones maintain immune tolerance against benign antigens such as non-inherited maternal polymorphic MHC and food antigens during gestation [2,3]. Despite the presence of these suppressive mechanisms, fetal T cells are capable of responding to foreign antigens [3]. After birth, newborns face the challenge with the sudden transition from a pathogen-free intra-uterine environment to an environment with an abundance of pathogens and increased risk of developing infectious disease. Due to lack of antigenic experience, neonates mainly depend on maternal antibodies and innate immune defensive mechanisms such as complement and phagocytes, although the anti-inflammatory phenotype of the innate immune system also contributes to limited defense [4,5,6]. This unique phenotype of innate immune cells likely plays an important role in the generation of host response against vaccines [7]. Pathogens that can cause significant morbidity in fetuses and neonates include *Haemophilus influenzae*, group B streptococci, *Escherichia coli*, herpes simplex virus (HSV), cytomegalovirus (CMV), varicella-zoster virus (VZV), respiratory syncytial virus (RSV), *Toxoplasma gondii*, *Listeria monocytogenes* and Candida species [4]. The primary mechanisms of host response against these pathogens are T cell- and antibody-mediated resistance [8]. Antibody-mediated host responses induced either by natural immunity or vaccines involve T cell-independent (TI) or T cell-dependent (TD) immune responses [9,10].

In this review, we discuss a range of immunobiological pathways participating in response to vaccines during early age. These factors span from placentally transferred maternal antibodies targeting pathogens to cells of innate and adaptive immune systems. We highlight the unique features of neonatal and infant immune systems that are responsible for the notoriously weak vaccine responses in this population. We also introduce emerging adjuvants that are tested in animal models and clinical studies to boost the immune response to vaccines intended for early age. Our review mostly covers the findings reported to PubMed^®^ during the last decade, although we also provide historical context when appropriate. The topics subjected to PubMed^®^ search were author-directed.

## 2. Age-Specific Immune Responses to Vaccines

### 2.1. Maternal Antibodies and Vaccine Responses

Maternally derived antibodies, passively transferred via the placenta (primarily IgG) and breast milk (primarily secretory IgA), are crucial for protecting newborns from infectious diseases early in life. The recognition of the beneficial effect of maternally transferred antibodies led to adoption of maternal immunization practices [11]. Vaccines against diphtheria, tetanus and RSV are examples of vaccines administered during pregnancy to prevent infections in newborns. Efficacy of existing vaccines given during pregnancy and other vaccines under development are beyond the scope of this review. However, we will discuss the impact of maternal antibodies on infants’ responses to pediatric vaccines because the transferred antibodies can present a significant clinical challenge by suppressing the infants’ active humoral response to vaccines, a phenomenon known as maternal antibody-mediated interference (MAMI) [12,13]. This interference is multifactorial, with several mechanisms that blunt the infants’ ability to mount a robust immune response. The most significant of these is “epitope masking,” where maternal IgG binds to vaccine antigens, causing steric hindrance that prevents the antigen from engaging the B cell receptor (BCR) on infant B cells. This binding also facilitates the rapid opsonization and clearance of the vaccine antigen before it can be effectively recognized [14,15].

Furthermore, maternal IgG–antigen immune complexes can deliver a potent inhibitory signal by co-engaging the BCR alongside the inhibitory Fcγ receptor IIB (FcγRIIB) on the surface of neonatal B cells, which express this receptor at elevated levels [16,17]. This signal actively suppresses B cell activation, proliferation, and differentiation into antibody-producing plasma cells. The resulting reduction in antigen availability and blunted B cell activation indirectly impairs the development of T follicular helper (Tfh) cells, which are essential for the generation of long-lasting immunity, thus compounding the intrinsic developmental immaturities of the neonatal immune system [18].

The inhibitory effects of MAMI are directly proportional to the titer of maternal antibodies at the time of vaccination [19]. Transplacentally acquired IgG has a half-life of approximately 3 to 4 weeks, with concentrations waning substantially over the first 6 to 12 months [12,20]. Standard multi-dose pediatric schedules are deliberately designed as a prime-boost strategy to navigate this period. While early vaccine doses may be partially neutralized, subsequent booster doses are administered when maternal antibody levels have fallen below the inhibitory threshold, allowing for a robust secondary immune response that generates high-affinity antibodies and durable immunological memory [19]. For vaccines highly susceptible to MAMI, such as the live-attenuated measles vaccine, this interference is markedly pronounced and constitutes one of the reasons for delaying the administration of the first dose until 9 to 12 months of age [14].

### 2.2. T Cell-Independent Responses

**Figure 1:**
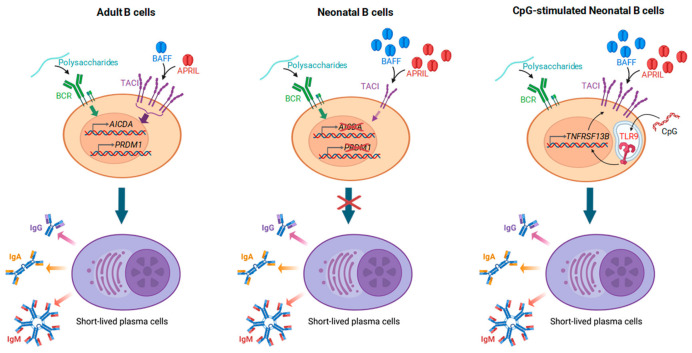
T cell-independent response to polysaccharide vaccines. The generation of a robust TI immune response is dependent on the coordinated integration of signaling cascades initiating from both the BCR and TACI. In the neonatal context, B cells exhibit a markedly diminished capacity to mount effective responses to polysaccharide-based vaccines due to the profound downregulation of TACI surface expression during this critical developmental window. Notably, the administration of CpG oligodeoxynucleotides has been demonstrated to partially circumvent this immunological limitation by upregulating TACI expression on neonatal B cells, thereby restoring their competence to participate in TI humoral immune responses.

Typically, polysaccharide antigens of encapsulated bacteria such as *Neisseria meningitidis*, *H. influenzae* and *S. pneumoniae* trigger a TI response whereas proteins induce a TD response. The repetitive multi-epitopes of polysaccharide antigens bind and cross-link BCRs [9] (Figure 1). However, cross-linking of BCRs alone is not sufficient to generate antibody responses against polysaccharides and a second signal is needed to activate B cells [9,21]. In both mice and humans, the physiological second signal is primarily mediated through transmembrane activator and calcium-modulating cyclophilin ligand interactor (TACI) expressed on B cells [22]. TACI is a receptor for TNF family cytokines B cell-activating factor of the tumor necrosis factor family (BAFF) and a proliferation-inducing ligand (APRIL) [22,23]. Both mice deficient in TACI and individuals with mutations in *TNFRSF13B* (TACI gene) respond poorly against polysaccharide vaccines [24,25]. Since the discovery of polysaccharide vaccines, it has been well established that these vaccines do not mount/elicit strong antibody responses in infants [26]. Studies in humans and mice revealed that B cell TACI levels are severely reduced at an early age, and in vitro stimulation of B cells from this population with BAFF or APRIL does not induce immunoglobulin secretion [27,28] (Figure 1). Underscoring the significance of low TACI expression on neonatal mouse B cells, CpG improves the immunogenicity of TI antigens in neonates by inducing the expression of TACI on B cells [28,29] (Figure 1). As observed in neonatal mice, adult XID mice with a mutation in Bruton’s tyrosine kinase gene (*Btk*) also do not respond to TI antigens [30]. Analysis of XID mice revealed that these mice also have severely reduced TACI expression on their B cells [31]. Diminished TACI expression plays an important role in the unresponsiveness of XID mice to TI antigens because, similar to neonatal mice, CpG treatment upregulates TACI expression on B cells in XID mice, and immunization of adult XID mice with CpG and NP-Ficoll largely restores antibody responses [31]. The fact that BCR signaling in adult mice, but not in neonatal mice or adult XID mice, induces upregulation of TACI expression indicate that immature BCR signaling during the neonatal period prevents the expression of TACI [28,31]. Importantly, CpG appears to bypass the BCR cross-linking requirement for TACI expression because treatment of neonatal wild-type or adult XID mice B cells with CpG increases TACI expression in the absence of BCR engagement and renders B cells from both the mice responsive to BAFF or APRIL-induced immunoglobulin secretion [28,31]. Collectively, the discovery of TACI and its role in B cell responses to TI antigens have helped unveil the immunobiological basis for weak responses to polysaccharide vaccines in infants [22].

### 2.3. T Cell-Dependent Responses

Unlike pure polysaccharide vaccines, the immune response to protein-conjugated polysaccharide vaccines as well as protein-based vaccines requires CD4+ T cell help [32]. The conversion of polysaccharides from TI to TD antigens by conjugation to proteins led to the development of *H. influenzae* type b (Hib) and multivalent *S. pneumoniae* conjugate vaccines, which are immunogenic in infants. These vaccines have led to a dramatic decrease in infant mortality and morbidity due to infections with Hib and *S. pneumoniae* [19]. However, like most other pediatric vaccines, both Hib and conjugate *S. pneumoniae* vaccines need to be administered three to four times during the first 15 months of life in order to elicit durable, long-lasting antibody responses [33]. Understanding how the same vaccines can elicit a protective immune response in adults after a single immunization, while infants need four doses to achieve the same outcome, remains a critical challenge in development of improved pediatric vaccines. The attributes associated with the developmental stages of cells involved in germinal center (GC) reaction likely play an important role in the unique phenotype of early-age immune responses to infections and vaccines.

A consequence of the increase in the number of conjugate vaccines and the valency of conjugate vaccines is the emergence of the phenomenon termed “carrier-induced epitopic suppression.” Most conjugate vaccines administered to children share the same carrier proteins, such as Tetanus Toxoid (TT), Diphtheria Toxoid (DT), or the genetically detoxified mutant of the DT (CRM197). As infants receive sequential doses (e.g., at 2, 4, and 6 months) of these vaccines, cumulative exposure rapidly builds strong anti-carrier immunity [34,35]. Consequently, pre-existing anti-carrier antibodies could mask polysaccharide epitopes, and the rapidly expanding carrier-specific B cells also outcompete polysaccharide-specific B cells for limited T-helper cell support [36,37,38]. Recognition of this phenomenon stimulated efforts to test alternative/novel carrier proteins in infant vaccines [39,40,41].

### 2.4. CD4+ T Cells

Recently emigrated thymic naïve CD4+ T cells dominate the T cell compartment of neonatal secondary lymphoid organs [42,43]. The ratios of different CD4+ T cell subsets (naïve, central memory, effector memory, and tissue-resident effector memory) in neonates differ from those in adults and are equipped with innate-like features such as expression of CXCL8, signaling through complement receptor and toll-like receptors (TLRs) that provide rapid protection against infections but confer limited immune memory [43]. In adults, the CD4+ T cell response is directed towards providing protection against invading pathogens and building long-lasting protection through the generation of memory cells. CD4+ T cell responses are mediated by professional antigen-presenting cells (APCs), B cells, and other T cell subsets through direct interaction and cytokine signaling. Once activated, CD4+ T cells secrete cytokines and differentiate into different CD4+ T cell subsets. Unlike the adult cells, the neonatal CD4+ T cell response is heavily biased towards Th2 (producing IL-4, IL-5, IL-13) and away from Th1 (producing IFN-γ, TNF-α) response, a skewing that is both intrinsic to the T cells and driven by the neonatal innate cytokine milieu [44,45,46]. In neonates, Th2-skewed response is further enhanced by IL-4 induced apoptosis of Th1 cells [47]. Bulk-RNA seq studies have demonstrated that the gene expression profile of neonatal CD4+ T cells is different than their adult counterparts. Pathway analysis of differentially expressed genes in naïve cord blood CD4+ T cells shows increased expression of cell-cycle-related genes and retention of a regulatory T cell (Treg) signature [48,49]. Additionally, lower expression of genes involved in T cell receptor (TCR) signaling (TCRα, TCRβ, LAT, LCK) leads to decreased TCR/CD28 signaling and low expression of activation marker CD69 [50]. Partly due to the weak interactions between APCs and CD4+ T cells, neonatal CD4+ T cells likely receive suboptimal stimulation signals, which results in an altered cytokine production profile, which eventually leads to decreased antibody responses [33]. Nevertheless, neonatal CD4+ T cells respond to TCR/CD28 stimulation by proliferating more than the adult cells and overexpressing the cell cycle genes, but their overall response is characterized as innate-like and tolerogenic [51].

**Figure 2:**
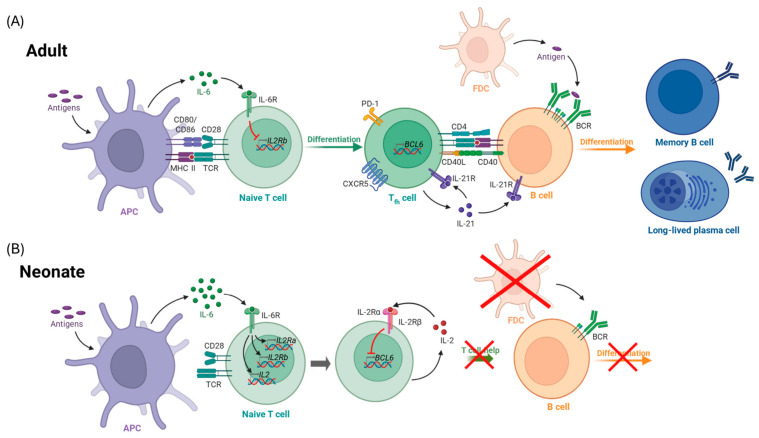
T cell-dependent response. (**A**) In vaccinated adult mouse, IL-6 secreted by APCs plays a pivotal role in orchestrating Tfh cell differentiation through the transcriptional induction of key surface molecules including CXCR5 and PD-1 as well as the promotion of IL-21 production. Concomitantly, IL-6 signaling suppresses the expression of IL-2Rβ, thereby attenuating IL-2-mediated signaling in differentiating T cells. Within the GC microenvironment, GC B cells that receive cognate T cell help delivered via CD40L and IL-21 from Tfh cells in conjunction with antigen capture by FDCs undergo terminal differentiation into long-lived plasma cells or memory B cells. (**B**) During neonatal immune response, however, this fine-tuned cascade is substantially compromised at multiple levels. Neonatal APCs exhibit markedly reduced surface expression of MHCII and co-stimulatory molecules CD80/CD86, rendering them poorly equipped to drive effective Tfh cell differentiation. Paradoxically, neonatal immune cells simultaneously overproduce IL-6, which, rather than promoting Tfh differentiation, aberrantly upregulates IL-2Rα, IL-2Rβ, and IL-2 cytokine production in CD4^+^ T cells. The resultant amplification of IL-2 signaling exerts an inhibitory effect on Tfh cells by suppressing the expression of BCL6, the master transcriptional regulator of the Tfh cell lineage. Consequently, neonatal B cells are deprived of adequate T cell-derived help necessary for productive GC reactions. This deficit is further compounded by delayed FDC maturation in neonates, which impairs the efficient capture and presentation of antigens to GC B cells. Collectively, these interconnected immunological shortcomings converge to significantly attenuated humoral immune responses during the neonatal period.

A closer look at the events that govern the development of TD responses involving the GC reaction indicates that neonatal GC response has fundamental differences compared to well-studied adult response. In adults, immunization results in the recognition of peptides on DC MHCII molecules by CD4+ T cells in the T cell zones of secondary lymphoid organs [18] (Figure 2A). This interaction leads to the expression of CXCR5 and ICOS which define the pre-T follicular helper (Tfh) cell signature [18]. Pre-Tfh cells expressing CXCR5 relocate to B cell follicles, where the CXCR5 ligand CXCL13 is produced. After the relocation of pre-Tfh cells to B cell follicles, the increased expression of molecules such as PD-1, SAP and the transcription factor, BCL6 completes the formation of fully mature Tfh cells and GCs. Multiple rounds of interactions between Tfh cells and antigen presenting GC B cells lead to the generation of high-affinity antibody-producing plasma cells and memory B cells [52] (Figure 2A). In addition to the contact-dependent interaction between APCs, Tfh cells and GC B cells, cytokines also participate in TD vaccine responses. Following APC:CD4+ T cell interactions and the initiation of pre-Tfh formation, IL-6 produced by APCs strongly augment adult mouse Tfh cell generation by inducing CXCR5 expression and IL-21 production [53,54,55] (Figure 2A). IL-21, in turn, stimulates the expression of BCL6, the key Tfh cell transcription factor [56,57]. IL-21 produced by Tfh cells is also important in the generation of GC B cell formation and proliferation [55,58]. Contrasting the Tfh cell-promoting activities of IL-6 and IL-21, IL-2 prevents excessive Tfh activity by inhibiting BCL6 expression [59]. Curtailing the inhibitory action of IL-2, IL-6 blunts IL-2-mediated STAT5 phosphorylation by downregulating the expression of IL-2Rβ [60] (Figure 2A).

Underscoring the importance of the development of Tfh cells in response to infections and TD vaccines, studies in mice, nonhuman primates (NHPs) and humans show that early age Tfh cell development is restricted compared to that in adults, and the diminished Tfh cell response correlates with weak antibody responses [61,62,63]. Mouse adoptive transfer experiments demonstrated that both neonatal CD4+ T cell intrinsic properties and neonatal mouse environment play a role in weak Tfh and GC B cell responses following immunization [62]. For example, the inhibitory cytokine, IL-2 is produced substantially more in immunized neonatal mice Tfh cells [64]. More detailed analyses indicated that vaccination induces significantly higher IL-6 production in splenic cells of neonatal mice compared to those of adult mice [65] (Figure 2B). Surprisingly, unlike in adults, the elevated IL-6 does not lead to the expansion of Tfh cells in neonates. Instead, IL-6 specifically suppresses neonatal Tfh cell development by increasing Tfh cell IL-2 production and the expression of its receptors IL-2Rα and IL-2Rβ [65,66] (Figure 2B). Underscoring the opposing role of IL-6 in the two age groups, immunization of IL-6-deficient neonatal mice results in improved Tfh cell generation, whereas IL-6 deficiency blunts Tfh cell development in immunized adult mice. Consistent with weak Tfh cell development, immunized neonatal mice exhibit a higher splenic Foxp3+ T follicular regulatory helper (Tfr) cell-to-Tfh cell ratio compared to adult mice and this ratio further increases when mice are immunized with a vaccine containing IL-6 [65,66].

An additional possible mechanism contributing to the blunted Tfh development in neonates may be due to heightened IL-7-mediated suppression. Together with IL-2, IL-7 is also shown to suppress BCL6 and CXCR5 expressions in Tfh cells through STAT5 [67]. Since IL-7 activity on T cells is reported to be more potent in early age, Tfh cell development may be restricted as a result of STAT5 activation [68].

### 2.5. GC B Cells

Following immunization, GC B cells undergo somatic hypermutation and affinity maturation of their antibodies before differentiating into plasma cells and memory B cells [52]. The activation of GC B cells in immunized adult mice depends on interactions between newly arrived Tfh cells and antigen-specific B cells residing in the B cell zone [18] (Figure 2A). Due to this requirement, insufficiencies in both neonatal Tfh cells and B cells contribute to restricted GC B cell formation in immunized neonates (Figure 2B). For example, subdued Tfh cell formation by itself is a limiting factor for GC B cell activation. Since IL-21 produced by Tfh cells acts directly on GC B cells to activate them, the consequence of diminished Tfh cell development is reduced proliferation, differentiation, and maintenance of GC B cells [58]. Besides IL-21, GC B cells receive additional activation signals from Tfh cells through direct contact between CD40 on B cells and CD40L expressed on Tfh cells [69] (Figure 2A). However, neonatal CD4+ T cells are known to be deficient in optimum CD40L expression upon activation [70] (Figure 2B). As a result, the activation of neonatal GC B cells remains further subdued.

B cells residing in the light zone (LZ) of GC capture antigens from follicular dendritic cells (FDCs) through their BCRs [52]. BCR-mediated antigen capture, cross-linking, signaling and internalization of antigen are essential functions of B cells before presenting peptides to Tfh cells in GC [52]. In neonatal mouse spleen, the differentiation of FDCs is delayed until after the mice are weaned [71]. This delay likely plays a role in insufficient antigen capture by FDCs resulting in diminished antigen availability for GC B cells. In addition to this limited antigen availability, BCR cross-linking does not lead to proliferation of neonatal B cells [72]. This inability to proliferate stems from the fact that there are substantial differences between adult and neonatal BCR signaling events [73]. However, the unresponsiveness of neonatal B cells to BCR cross-linking can be overcome by second signals such as IL-1β and CpG [74].

In addition to their dampened proliferative response to BCR cross-linking, IL-10-producing CD43+ B1 cells constitute 1/3rd of neonatal mouse splenic B cells [75]. In adult mice only 2% of splenic B cells are B1 cells. Interestingly, unlike in adults, neonatal splenic non-B1 cells (CD19 + CD43-) also contribute to total IL-10 production [76]. In non-B1 cells, BCR-induced IL-10 production is dependent on initial IL-6 secretion which induces IL-10 in an autocrine and paracrine fashion [76]. Since both human and mouse IL-10 producing B cells create a suppressive environment [77,78], abundance of IL-10 producing B cells in neonatal spleen likely participates in subduing vaccine responses.

### 2.6. Innate Immune Cells and GC Reaction

Like the adaptive immune system, the neonatal innate immune system also manifests constrained response to environmental signals. The main elements of innate immune system include physical barriers and cells such as phagocytes (neutrophils, macrophages), DCs, innate lymphoid cells, myeloid-derived suppressor cells and natural killer cells that recognize molecular patterns common to pathogens and respond rapidly to contain pathogens [79]. Among these cells, DCs are the primary APCs that initiate CD4+ T cell response [80]. Total DC numbers in neonatal lymphoid tissues are reduced compared to adults, and their subset distribution undergoes dynamic postnatal changes [44,81]. In murine models, conventional DC type 1 (cDC1) populations predominate early in life, whereas cDC2 populations expand with age [44]. Expression of canonical subset markers, such as CD8α on cDC1, is developmentally delayed. Characterization of early age DC functions mostly depend on analyzing responses following stimulation with bacteria or pathogen-associated molecular patterns (PAMPs) [82]. These analyses clearly show that the responses of newborn mice and human DCs differ from those of adult DCs [83,84,85]. Intrinsically, neonatal DCs are biased against Th1 polarization, mostly because they produce less IL-12p70 (a key Th1-driving cytokine) following TLR4, TLR3 or CD40 engagement [44,82,86] and epigenetic repression of IL12p35 transcription further contributes to this defect [85]. Neonatal DCs also express lower levels of MHCII and co-stimulatory molecules. For example, expression of MHCII, ICAM-1, CD80, CD86 in cord blood DCs are lower than those in adult cells after stimulation with TLR agonists LPS, poly(I:C), CpG and R-848 [82,85]. Distinct pattern of cytokine secretion following TLR agonist stimulation is another attribute of neonatal DCs [87]. The inability of neonatal DCs to produce IL-12 during antigen presentation may also be related to the fact that neonatal DCs secrete IL-10 more than the adult DCs do [45,82,88]. Since IL-10 can drive Treg cell formation, DC-produced IL-10 likely plays a role in restricting Th1 response by promoting Treg cells [89]. Neonate specific milieu also contributes to the blunted DC response to stimuli. Higher plasma concentrations of adenosine and specific microRNAs, such as Let-7g, can shape the activation of DCs in response to TLR stimulation, further modulating their function away from an adult-like pro-inflammatory state [90,91]. The unique in vivo environment of neonates is most notable in experiments where neonatal mice are immunized with tetanus toxoid-conjugated pneumococcal serotype 14 polysaccharide (PPS14-TT) with CpG. As indicated above, splenic cells from neonatal mice, including DCs, produce significantly more IL-6 compared to adult mice following immunization with PPS14-TT and, unlike in adults, IL-6 suppresses Tfh cell development [65]. When neonatal and adult innate cells are incubated in vitro with CpG, neonatal cells produce significantly more IL-6 compared to adult cells [92]. However, in contrast to its in vitro IL-6-inducing effect, and underscoring the unique immunomodulatory properties of neonatal mice, administration of CpG with PPS14-TT in neonatal mice decreases the percentage of IL-6-producing splenic cells, including DCs and macrophages [65]. Additional neonate-specific in vivo inhibitory factors include maternal milk-derived serum lipids, which dictate the phenotype of neonatal macrophages in mice. These lipid species lead to formation of lipid droplets in macrophages and convert them to an immunosuppressive phenotype that resemble tumor-associated macrophages [93]. Whether neonatal serum lipids regulate other immune cells remains to be explored.

### 2.7. Adjuvants

Recognizing the vulnerability of infants to infections until they complete the three to four doses of routinely administered pediatric vaccines, a spectrum of adjuvants has been tested to improve pediatric vaccine efficacy [33]. The majority of these studies have been conducted in animal models, although some adjuvants have also been evaluated in pediatric clinical trials. Most of the existing pediatric vaccines contain aluminum salts (alum) as adjuvants (Table 1). Although alum’s exact mechanism of action is still debated [94] and they tend to drive Th2 responses without enhancing Tfh cells, they are proven to be suitable for vaccines where antibody mediated protection is desirable [95]. An additional benefit of alum is decreasing inherent toxicity of vaccines such as meningococcal outer membrane vesicles [96]. The discovery of TLR agonist PAMPs as powerful activators of adaptive immunity prompted research into using them as vaccine adjuvants, including for pediatric populations [97,98,99]. For example, the squalene containing oil-in-water adjuvant MF59 has been shown to increase the immunogenicity of influenza vaccines in infants 6 months and older and to elicit higher antibody responses [100] (Table 1). Similarly, the AS01 (includes MPL and quillaja Saponaria 21) adjuvanted malaria RTS, S vaccine has been shown to be immunogenic in infants as young as 6 weeks old [101] (Table 1).

Soon after the discovery of Tfh cells and their central role in dictating TD immune responses to vaccines in adult mice, the inability of neonates to mount Tfh cell responses to vaccines was recognized as an immunobiological limitation responsible for weak antibody responses to pediatric vaccines [62,102]. This recognition was accompanied by testing different adjuvants to improve Tfh cell and GC reaction in neonatal mice. The TLR9 agonist type B CpG was the first adjuvant to show significant improvement in Tfh cell development in neonatal mice [62] (Table 1). Similarly, a liposome-incorporated dimethyldioctadecyl-ammonium (Mincle agonist) influenza hemagglutinin vaccine was shown to induce the development of Tfh cells accompanied by elevated antibody responses [103].

There has been considerable interest in investigating TLR7/8 agonists as adjuvants in early age mostly because TLR7/8 activity on human infant APCs, B cells and T cells is comparable to that in adults’ [104,105]. The CAF08 adjuvant, which is composed of TLR7/8 agonist and the C-type lectin receptor Mincle, augments antigen specific CD8+ and Th1-skewed CD4+ T cell responses in neonatal mice and leads to higher antibody responses when injected with RSV pre-fusion antigen [100] (Table 1). Interestingly, the increase in antibody responses against RSV pre-fusion antigen was not accompanied by an increase in Tfh cell development, although IFN-γ-secreting Th1 cell responses were enhanced. Nonhuman primate studies also demonstrated improved antibody responses in animals less than one week old when immunized with influenza antigens together with conjugated or unconjugated forms of TLR7/8 agonist R848 [106,107,108,109,110]. Reflecting the broad expression profile of TLR7/8 in human newborn cells, an R848-containing influenza vaccine activated draining lymph node B cells in neonatal NHPs [111]. In another NHP study, immunization of newborns with the 13-valent pneumococcal vaccine (Prevnar13) resulted in increased antibody responses when formulated with the TLR7/8 agonist 3M-052 compared to the vaccine alone [112]. Similarly, immune response to a licensed acellular pertussis vaccine was substantially improved in neonatal mice when the vaccine was adjuvanted with a TLR7/8 agonist [113].

Typically, when adjuvants are tested in animal studies, the responses are compared to an aluminum-based counterpart or a placebo group. In a more comprehensive evaluation, Pind and colleagues compared the immune response in neonatal mice using a TT-conjugated pneumococcal polysaccharide 1 (Pnc1-TT) adjuvanted with LT-K63 (non-toxic heat labile *Escherichia coli* endotoxin), mmCT (non-toxic multiple-mutant cholera toxin), MF59, IC31 (combination of antibacterial peptide KLK and TLR9 agonist, ODN1a), CTB-CpG (combination of CpG and the non-toxic B subunit of cholera toxin) and Alum (potassium aluminum sulfate) molecules [114] (Table 1). Serum antibody measurement indicated that the CTB-CpG vaccine yielded comparable IgG and IgM responses against Pnc1 as Pnc1-TT alone or Alum-adjuvanted Pnc1-TT while all other adjuvants augmented both antibody production and GC development [114]. This study also showed that the improved GC formation was accompanied by enhanced FDC maturation with LT-K63, mmCT, MF59 and IC31 adjuvants. It is not clear why one of the two TLR9 agonist-containing adjuvants elicited GC formation while the other one did not, even though both augmented IgG and IgM antibodies against the vaccine antigen. One possible explanation is that CTB-CpG induces antibody responses through a TI pathway, as was observed in neonatal mice that developed augmented antibody responses to the TI antigen NP-Ficoll when the antigen was injected with CpG [28].

**Table 1:** Vaccine adjuvants tested in neonatal models.

Vaccine Adjuvant	Mechanisms Elucidated by Adult Studies	Outcomes Found in Neonatal Studies	References
Aluminum salts (Aluminum potassium sulfate, Aluminum hydroxide, Aluminum phosphate, and Aluminum hydroxide + magnesium hydroxide)	The “depot effect” holding the vaccine antigen at the injection site for a prolonged period and triggering the NLRP3 inflammasome in immune cells to produce vital inflammatory signals.	Induction of Th2-type T cell and antibody responses	[94,97]
MF59 (oil-in-water emulsion containing squalene, Tween 80, and Span 85)	Acute induction of inflammatory cytokines and chemokines to recruit various innate immune cells (monocytes, neutrophils, and eosinophils) and APCs at the injection site, triggering the differentiation of recruited monocytes into DCs, and induction of non-harmful apoptosis of DCs through TLR-independent MyD88 signaling pathway.	Induction of robust neutralizing antibody production and expansion of memory B cell pool	[97]
AS01 (MPL, QS-21 and liposome)	MPL activates immune cells via TLR4; QS-21 activates NLRP3 inflammasome with subsequent IL-1β/IL-18 release while facilitating antigen uptake and presentation by APCs; the cholesterol in the liposome serves as a delivery scaffold while suppressing the inherent hemolytic potential of QS-21.	Induction of the sustained robust Th1-biased immune response	[100]
Type B CpG (synthetic microbial-mimicking DNA molecules)	Induction of B cell activation, DC maturation, and proinflammatory cytokine secretion via TLR9.	Enhancing humoral responses by increasing Tfh and GC B cell development	[61]
CAF01 (Mincle agonist)	Triggering the C-type lectin receptor Mincle to increase pro-inflammatory cytokine production.	Enhanced antibody responses by driving robust Tfh and GC B cell responses; robust Th1/Th17 cellular immunity	[102]
CAF08 (Mincle agonist, synthetic TLR7/8 agonist and liposome)	Synergistically activating TLR7/8 and C-type lectin receptor Mincle.	Induction of robust Th1 and CD8+ T cell responses	[104]
3M-052 (synthetic TLR7/8 agonist)	Local tissue retention and robust Th1 cytokine production via TLR7/8.	Promoting robust GC formation, the development of long-lived plasma cells and memory B cells, protective IgG production, and opsonophagocytic killing activity	[111]
LT-K63 (detoxified mutant of the *E. coli* heat-labile enterotoxin)	Enhancing maturation of FDC network and GC formation.	Prolonged lifespan of plasma cells and persistent vaccine-specific antibody levels	[113]
mmCT (non-toxic multiple-mutant of cholera toxin)	Driving maturation of DCs and monocytes via NF-B pathway and enhancing GC formation.	Induction of robust Th1/Th17 responses; enhancing IgG and IgA responses	[96]
IC31 (antimicrobial peptide KLK and TLR9-activating sequence ODN1a)	KLK peptide creates a protective depot at the injection site, slowing down antigen release; ODN1a activates APCs via TLR9.	Induction of Th1 response; enhancing GC formation and vaccine-specific IgG response	[96]

Two vaccines developed in response to the coronavirus disease 2019 (COVID-19) pandemic were based on mRNA technology [115]. In addition to inducing CD8+ T cell response, severe acute respiratory syndrome coronavirus 2 (SARS-CoV-2) mRNA vaccines induce protective antibody responses against SARS-CoV-2. Both the vaccines were approved for infants 6 months and older. Among these vaccines, two 25 µg doses of mRNA-1273 were administered to children 6 to 23 months of age 28 days apart [116]. Neutralizing antibody levels measured 57 days after the second dose were comparable to those of young adults who were previously shown to be protected from COVID-19 following two 100 µg doses of mRNA-1273 vaccine [117]. The second SARS-CoV-2 mRNA vaccine, BNT162b2, was given as two 3 µg doses to children between 6 months and 2 years of age followed by a third dose at ≥8 weeks after the 2nd dose. Like the mRNA-1273 vaccine, efficacy of BNT162b2 was demonstrated by assessing statistical noninferiority of neutralizing antibody titers measured in young adults [118]. However, their efficacy against newborns and infants younger than 6 months remains unclear because they are approved for children 6 months and older [119]. Two recent animal studies have tested the immunogenicity of mRNA-based vaccines in NHP and mouse neonates [120,121]. In the NHP study, an mRNA vaccine expressing influenza HA protein was used [121], whereas, in mice, an mRNA vaccine encoding the receptor binding domain (RBD) of SARS-CoV-2 (mRNA-RBD) was evaluated [120]. In the mouse study, the mRNA-RBD vaccine elicited higher antibody responses with improved functional properties compared to recombinant RBD vaccine adjuvanted with AddaVax^TM^, a squalene-based oil-in-water nano-emulsion adjuvant. In the NHP study, mRNA-HA vaccine induced antibody responses accompanied by circulating IL-21-producing CD4+ T cells.

In addition to exploring a range of adjuvants to improve vaccine responses in early age, an alternative approach is to extend antigen availability for sustained stimulation of the host immune system [122]. This concept was demonstrated in newborn mice in a recent study where neonates receiving Alum-containing PPS14-TT vaccine in four doses (administered every two days) elicited significantly higher levels of antibodies against PPS14 compared to the same total amount of PPS14-TT administered as traditional bolus regimen [123].

Many adjuvants augment TD antibody responses through the induction of IL-6. For example, IL-6 secretion from splenic cells induced by lipid nanoparticles in mRNA as well as in protein-based vaccines is needed for the development of Tfh cells in adult mice [124]. Similarly, immunization studies in adult mice have shown that AS03, which is composed of α-tocopherol, squalene and polysorbate, stimulates Tfh cell generation and increases antibody production by inducing IL-6 secretion [125]. Other adjuvants that are shown to improve Tfh cell responses by inducing IL-6 are CpG, water-in-oil-based adjuvants and LPS [126,127,128]. As discussed above, adjuvants that induce IL-6 in vivo should be avoided in neonates because IL-6 suppresses Tfh cell development in neonatal mice [65]. Since in vitro IL-6-inducing properties of CpG on neonatal splenic cells is completely opposite of its in vivo activity [92], candidate adjuvants need to be carefully investigated for in vivo IL-6 production without relying on their in vitro stimulation results.

### 2.8. Limitations Associated with Murine Data

Insights into innate immune priming and age-specific immune characteristics derived from murine neonatal studies have informed our understanding of analogous processes in human infants. However, the translational relevance of these findings must be interpreted within a framework that accounts for fundamental biological differences between the two species [129,130]. One of the most noticeable differences is the overall pace of immune system maturation and lymphoid organ development. At birth, the murine spleen lacks a well-defined marginal zone, GC responses are markedly attenuated, and full splenic architectural maturity is not achieved until the second or third postnatal week [131,132]. In contrast, in human infants lymphoid organogenesis is considerably more advanced at term; nevertheless, the immune system remains functionally immature at birth and requires years to reach full maturity [133,134]. Consequently, the timeline of postnatal immune calibration in humans does not map linearly onto the compressed postnatal window observed in mice. Beyond developmental timing, additional interspecies differences further complicate direct comparisons. These include divergences in the relative proportions of innate immune cell subsets—such as the neutrophil-to-lymphocyte ratio—as well as differences in TLR expression profiles and commensal microbiome composition [130,135,136,137]. Additionally, the mechanism and timing of maternal antibody transfer differ substantially between the two species. In humans, transplacental IgG transfer via the neonatal Fc receptor (FcRn) occurs predominantly during the third trimester, such that full-term neonates are born with robust maternally derived humoral protection [138]. In mice, only a modest fraction of maternal IgG is transferred transplacentally via FcRn during late gestation; a substantial proportion is instead delivered postnatally through breast milk absorbed across the neonatal intestinal epithelium [139,140,141]. As a result, at birth murine neonate is comparatively hypogammaglobulinemic relative to its human counterpart [142]. Taken together, these distinctions have direct implications for modeling vaccine-induced immunity in neonatal systems and underscore the importance of interpreting murine neonatal data with appropriate caution before drawing firm translational conclusions relevant to pediatric vaccine development or neonatal immunotherapy.

## 3. Conclusions

The suppressive and tolerogenic phenotypes of both the innate and adaptive immune system of neonates [143] present as a developmental gap hampering vaccine efficacy that leaves infants vulnerable to infectious diseases [33]. This fundamental distinction from the adult immune system is characterized by impaired T cell-independent responses to polysaccharide vaccines [22,26], a strong Th2 bias [44,45,46], and restricted development of Tfh and GC B cells [61,63,144], which are all crucial for generating robust and long-lasting immunity [52]. The opposing role of cytokines like IL-6 in neonates versus adults, suppressing rather than promoting Tfh cell development, exemplifies the unique immunological landscape of early life and underscores why adult vaccination strategies may not be appropriate for vaccines intended for infants [65,66].

The discovery of an inhibitory activity of IL-6 on murine neonatal Tfh cells also underscores the need to further investigate the molecular and cellular pathways governing neonatal GC and Tfh cell responses to vaccines. There is also a pressing need to extend these studies to human cells since there are notable differences between murine and human Tfh cell development and maintenance. For example, in humans IL-6 acts in concert with IL-12 and Activin A to drive the differentiation of Tfh cells [145]. Whether IL-6 or other cytokines exhibit inhibitory function on human neonatal Tfh cells also remains to be seen. Identification of these unique properties will likely help in devising novel strategies tailored to improve early age vaccine responses. A detailed understanding of the mechanisms of GC development, including both inhibitors and enhancers, can also aid in assessing factors that can impact vaccine responses. For example, both mouse and human immunization studies have shown that antibiotic use prior to immunization significantly reduces vaccine-induced antibody production through the elimination of beneficial bacteria belonging to Bifidobacterium species [146,147].

Several promising adjuvant strategies are emerging to overcome the challenges presented by the early age immune system. Adjuvants that can steer the neonatal immune system away from its default Th2-biased and tolerogenic state towards Th1 and Tfh cell responses are of particular interest. Toll-like receptor agonists, such as CpG and R848, have shown considerable promise in preclinical models by enhancing Tfh cell development and antibody production [62,106,107,108,109,110]. Furthermore, novel vaccine platforms, such as mRNA vaccines [120,121], and alternative delivery strategies that ensure sustained antigen availability [123] are exciting avenues that have demonstrated the potential to induce more potent and durable immune responses in the neonatal setting. However, despite these promising developments there is a pressing need for detailed mechanistic studies to understand precisely how different adjuvants function within the unique neonatal in vivo environment. Investigating the long-term effects of early-life adjuvant exposure on immune system programming is critical to ensuring both safety and efficacy, as some adjuvants can “train” the immune system for future responses [148]. For example, exposure to specific adjuvants during early age influences the type of CD4+ T cell response later in life. In addition to eliciting Th2 response in neonates, aluminum-based adjuvants also program the mouse immune system to enhanced Th2-skewed response to vaccines in adulthood [148]. Conversely, immunizing neonates with a Th1-polarizing CpG adjuvant during early age appears to train the DCs to stimulate Th1 response later in life. The degree and context of adjuvant-mediated imprinting on immune system during early age warrants further studies to better understand the beneficial as well as potential adverse outcomes of early age vaccination.

Future research must also focus on identifying reliable biomarkers that can predict vaccine immunogenicity in infants, which would accelerate the development of new and improved pediatric vaccines. Furthermore, the influence of external factors, such as maternal antibody transfer, breastfeeding [93], and the infant microbiome [146,147], on vaccine responses require further investigation to develop vaccination strategies tailored to the individual infant.

Addressing these immunobiological hurdles can potentially have profound clinical implications for global infant health. Developing vaccines that are effective from birth would meaningfully reduce the burden of neonatal infectious diseases [4,19]. Closing the window of vulnerability in early life [143] can help reduce infant morbidity and mortality, limit hospitalizations, and alleviate the immense strain on healthcare systems, particularly in low-resource settings. Ultimately, a deeper understanding of neonatal immunology is paramount to designing new generation of pediatric vaccines that can provide robust and long-lasting protection in infants.

## Data Availability

No new data were created or analyzed in this study. Data sharing is not applicable to this article.
